# Severe Hyponatremia as a Complication of Sepsis: A Case Report

**DOI:** 10.7759/cureus.79648

**Published:** 2025-02-25

**Authors:** Logan A Goddard, Adrienne Clermont, Mark Supino

**Affiliations:** 1 Emergency Department, University of Miami Miller School of Medicine, Miami, USA; 2 Emergency Department, Jackson Memorial Hospital, Miami, USA

**Keywords:** case report, emergency medicine, hyponatremia, sepsis, syndrome of inappropriate secretion of antidiuretic hormone (siadh)

## Abstract

Severe infections such as pneumonia can cause hyponatremia. This phenomenon is widely attributed to the syndrome of inappropriate antidiuretic hormone secretion (SIADH), although the mechanism is not fully understood. Healthcare providers must identify and treat the cause of low sodium correctly to prevent elevated morbidity and mortality. This report describes an uncommon case of severe hyponatremia in a 26-year-old male with no known medical history and no risk factors for immunosuppression. He presented with worsening body pain and shortness of breath, and he noted an ulcerated lesion on his lower back that had drained purulent fluid in the past week. Laboratory studies revealed severe hyponatremia, with a sodium level of 117 mEq/L, and chest X-ray depicted bilateral interstitial opacities concerning for pneumonia. The patient was started on normal saline, empiric antibiotics, and bilevel positive airway pressure in the emergency department. He was admitted to the intensive care unit (ICU) and subsequently required intubation. After a prolonged ICU course, the patient eventually made a full recovery with no residual deficits. Our report demonstrates an uncommon case of severe hyponatremia in a young, previously healthy patient. He presented with alarmingly low sodium levels, likely secondary to SIADH in the setting of sepsis due to skin abscess and pneumonia. The severity of the patient’s hyponatremia was a fundamental factor in identifying the critical nature of his disease and the need for expeditious treatment. Familiarity with appropriate management of hyponatremia is crucial for emergency physicians.

## Introduction

Hyponatremia, or low serum sodium, is a well-documented complication of severe infection, particularly in pneumonia [1,2]. While the precise mechanism is not completely understood, decreases in sodium concentration in the setting of infection are frequently attributed to the syndrome of inappropriate antidiuretic hormone secretion (SIADH). It is critical for providers to recognize and correct sodium levels appropriately, as hyponatremia is associated with increased mortality and morbidity, and inappropriate treatment can lead to death or severe disability [3]. The underlying cause of infection, and any concomitant illnesses, must also be addressed. Many cases of SIADH occur in patients with predisposing factors such as immunosuppressant medications, chronic illness, malnutrition, or old age. Unlike these cases, this report highlights a unique case of severe hyponatremia in a young, previously healthy patient with no known risk factors and contributes to the literature on emergency department (ED) management of hyponatremia in severe sepsis.

## Case presentation

A 26-year-old male with no known past medical history presented to the ED with three days of worsening myalgias and generalized body pain. He noted that the pain started in his legs but had now spread to his entire body, and he had begun feeling short of breath in the last 24 hours. He denied sick contacts, recent travel, or recent illnesses. On review of systems, he noted a “pimple” on his lower back. He was unsure of how long the lesion had been present but stated that it had spontaneously drained some purulent fluid in the past week. The patient revealed smoking tobacco and marijuana daily but denied any other substance use. He denied any prior history of HIV or other risk factors for immunosuppression.

Vital signs on arrival were as follows: oral temperature 36.6 degrees Celsius, heart rate 135 beats per minute, blood pressure 150/94 mmHg, respiratory rate 30 breaths per minute, and oxygen saturation 95% on room air. Physical examination was notable for a diaphoretic and toxic-appearing young man, in visible distress due to pain, along with heavy, rapid breathing with coarse crepitation bilaterally, a 2 cm non-fluctuant, well-circumscribed ulcerated lesion on the left buttock at the level of the sacrum, and a non-focal neurologic examination other than diffuse weakness secondary to pain.

**Figure 1:**
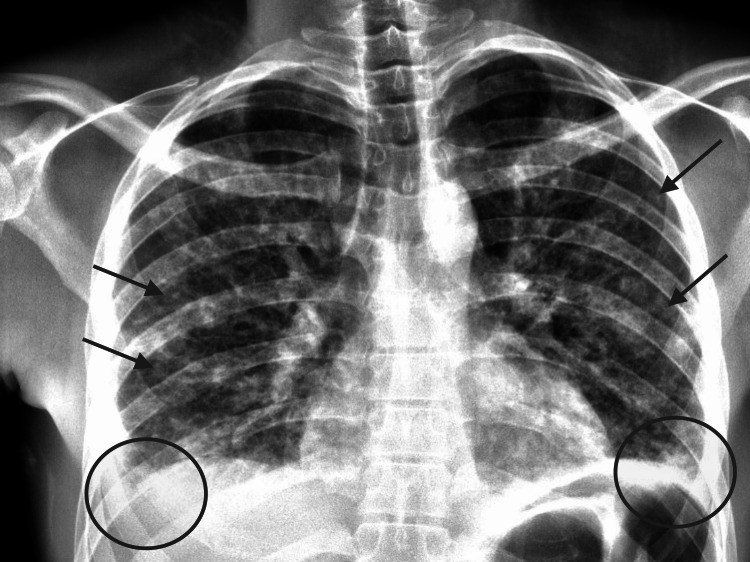
Patient’s chest X-ray at presentation Arrows indicate bilateral interstitial opacities. Circles indicate bilateral pleural effusions.

Diagnostic workup was begun, including venous blood gas, urine osmolality, serum osmolality, complete blood count, complete metabolic panel, and infectious respiratory panel. The patient’s initial venous blood gas was reassuring, though laboratory results revealed severe hyponatremia (Table 1). Serum osmolality was low at 242 mOsm/kg, and urine studies showed a urine sodium concentration of >20 mmol/L and urine osmolality of 563 mOsm/kg. Taken together, these results are indicative of SIADH [4]. Renal function was impaired with creatinine 1.7 mg/dL, indicative of acute kidney injury (AKI). Complete blood count was notable for a normal white blood count, however with significant bandemia of 21%, mild anemia, and significant thrombocytopenia. Other notable laboratory studies included an elevated lactic acid and elevated creatine phosphokinase. HIV test was negative. Viral respiratory panel was positive for coronavirus 229E. Chest X-ray showed bilateral interstitial opacities and bilateral pleural effusions concerning for pneumonia (Figure 1). Given the patient’s presentation with systemic signs of infection, respiratory distress, and severe hyponatremia, initial differential diagnoses included bacterial pneumonia, aspergillosis, *Legionella pneumonia*, viral pneumonitis, *Mycobacterium *tuberculosis, and other infectious causes of SIADH. Intravenous normal saline was administered with serum sodium checks ordered every two hours to begin cautiously correcting the hyponatremia. Azithromycin and ceftriaxone were started for the treatment of suspected community-acquired pneumonia, and the patient was placed on bilevel positive airway pressure for respiratory support.

**Table 1:** Initial laboratory investigation values at ED presentation ED, emergency department

Parameter	Patient value	Reference range	Unit
pH	7.38	7.35 - 7.45	
CO_2_	44	33-35	mm Hg
HCO_3_	25	22-26	mm Hg
Na	117	135-145	mEq/L
K	3.4	3.5-5.0	mEq/L
Cl	83	95-105	mEq/L
Ca	1.03	8.4-10.2	mg/dL
Serum osmolality	242	>275	mOsm/kg
Urine Na	>20	<20	mmol/L
Urine osmolality	563	<100	mOsm/kg
Creatinine	1.7	0.7-1.3	mg/dL
White blood cells	5,000	4,000-11,000	/mm^3^
Hgb	13.1	13.5-17.5	g/dL
Platelets	96,000	150,000-400,000	/mm^3^
Lactic acid	2.6	0.5 - 2.2	mmol/L
Creatine phosphokinase	1585	10-120	mcg/L

**Figure 2:**
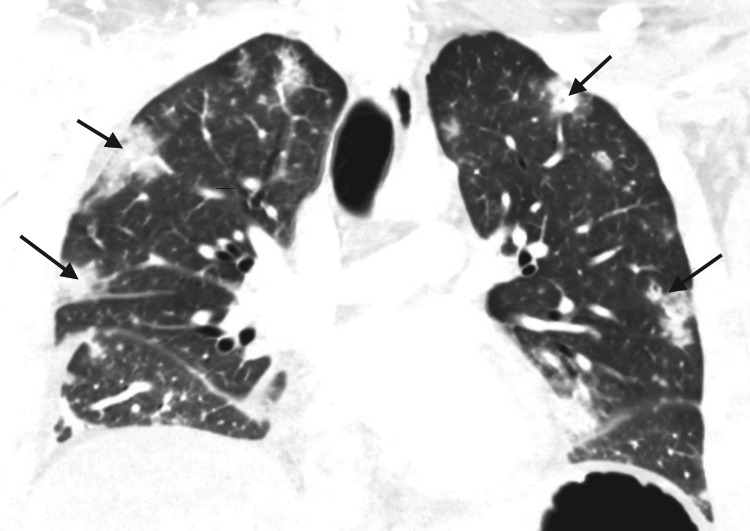
Patient’s chest CT at presentation Arrows indicate bilateral cavitary lesions

Around 10 hours after initial presentation, the patient was admitted to the intensive care unit (ICU). The patient's serum sodium levels gradually increased as normal saline was continuously administered, increasing by 9 mEq/L in 24 hours (Table 2). This increase is slightly above the recommended increase of 8 mEq/L in 24 hours, but it is near the ideal value and presents low risk of osmotic demyelination syndrome (ODS) [5]. The patient's respiratory status continued to decline; arterial blood gas results several hours after ICU admission (while on high flow nasal cannula) were as follows: pH 7.2, pO_2_ of 79 mmHg, pCO_2_ of 62 mmHg, and HCO_3_ 24 mmol/L. He was intubated within a day of admission, and his Sequential Organ Failure Assessment (SOFA) score at this time was 7, indicating elevated mortality risk. Computed tomography (CT) chest with contrast showed multifocal airspace opacities and bilateral cavitations, concerning for cavitary multifocal pneumonia or possibly septic emboli (Figure 2). CT of the abdomen/pelvis showed filling defects in the bilateral common iliac veins and in the inferior vena cava, consistent with multifocal venous thrombosis. The patient was started on an IV heparin drip to treat his venous thromboses. Two-dimensional transthoracic echocardiogram with Doppler showed no cardiac abnormalities except tachycardia and trace pericardial effusion.

**Table 2:** Time since presentation versus serum sodium values

Time since presentation (hours)	Serum sodium (mmol/L)
0	117
2	119
6	121
12	122
24	126
36	128
48	137
60	140
72	141

Two days later respiratory, blood, and abscess wound cultures were all found to grow methicillin-sensitive *Staphylococcus aureus* (MSSA). Bronchoalveolar lavage galactomannan returned positive, indicating concurrent Aspergillus infection. Given the confirmed infectious source of MSSA with Aspergillus, antibiotic coverage was broadened to meropenem, vancomycin, and voriconazole. Four days after ICU admission, his serum creatinine decreased to 0.6, demonstrating resolution of his AKI.

Despite eunatremia and normal kidney function, the patient's respiratory status remained poor. He required paralysis and frequent prone positioning due to poor oxygenation, ultimately remaining intubated for 14 days before successful extubation, with discharge home six days later. Upon follow-up one month later, the patient reported that he had fully recovered to his baseline health status with no residual symptoms or deficits.

## Discussion

Sodium imbalances are a common problem for emergency physicians, with up to 10% of patients presenting with hyponatremia [6]. This disorder is particularly prevalent among patients with pneumonia and severe infections. Upon hospital admission, up to 27.9% of patients with pneumonia and 40.3% of patients with sepsis have hyponatremia [2,7]. Hyponatremia is defined as serum sodium <135 mEq/L, with severe hyponatremia defined as sodium <120 mEq/L [8]. While mildly hyponatremic patients are often asymptomatic, patients with severe hyponatremia most commonly present with complaints of nausea, confusion, or weakness but can progress to seizures and coma [6].

Low serum sodium can result from many mechanisms including infectious, renal, endocrine, metabolic, and nutritional pathologies. In the setting of severe infection, the most common culprits are sepsis, *Legionella pneumonia*, and HIV/AIDS [1]. The precise pathophysiology of hyponatremia in infection is incompletely understood but is widely attributed to SIADH [9]. Normally, physiological levels of antidiuretic hormone (ADH) are secreted by the posterior pituitary in response to serum hyperosmolarity. ADH binds to vasopressin receptors in the kidney to increase aquaporin insertion on the apical membrane of the collecting duct, thus increasing water transport into the cell. It is hypothesized that during an infection, elevated inflammatory cytokines increase ADH secretion to supraphysiologic levels, leading to hyponatremia [9]. The current standard to diagnose SIADH as the cause of hyponatremia is the Bartter and Schwartz criteria, first established in 1967 [4]. Bartter and Schwartz describe these criteria as serum osmolality <275 mOsm/kg, urine osmolality >100 mOsm/kg, urine sodium >20 mmol/L, euvolemia, and no other causes for hyponatremia [10]. Other possible mechanisms of low serum sodium in infection that were considered on the differential include a reset osmostat (hypovolemia induced by extrarenal sodium losses), decreased oral intake, or systemic vasodilation [10]. In this case, the patient met all four Bartter and Schwartz criteria: serum osmolality was 242 (<275 mOsm/kg), urine osmolality was 563 (>100 mOsm/kg), urine sodium was >20 mmol/L, the patient was euvolemic, and no other chronic diseases, medications, or other causes for hyponatremia were identified. Therefore, SIADH was the likely cause of his hyponatremia.

Moderate-to-severe hyponatremia upon hospital presentation is associated with poor outcomes, including increased mortality and longer hospital stay in patients with sepsis and community-acquired pneumonia [2,7]. Hyponatremic patients with pneumonia have also been found to have higher rates of ICU admission and mechanical ventilation [11].

Given the associated outcomes, prompt ED management of hyponatremia is critical. Initial steps for the emergency physician are dependent on the severity and chronicity of the hyponatremia [12]. However, in the setting of infection with severe hyponatremia, chronicity can be difficult to establish. If there is a documented drop in serum sodium >10mEq/L within the last 48 hours and the patient is symptomatic, guidelines suggest 150 mL 3% saline titrated over 20 minutes (1-2 mL/kg body weight) with a serum sodium re-check in 4 hours [13]. If the chronicity is unknown, treatment becomes more difficult as rapid correction of chronic hyponatremia by >12 mEq/L over 24 hours is a well-known cause of ODS [5]. While there is no randomized trial evidence to guide management in this scenario, conventional guidelines dictate use of 0.9% saline infusion to achieve a gradual correction of 8 mEq/L over 24 hours, with monitoring every 2 to 3 hours [14]. Providers may vary the precise rate of correction depending on the severity of patient symptoms and the perceived consequences of further prolonging the hyponatremia versus risks of ODS. In this case, the chronicity was unknown, and therefore 0.9% saline was infused with serum sodium checks every 2 hours and an ideal target of 8 mEq/L increase over 24 hours. The actual achieved correction rate was appropriate, with a total serum sodium increase of 9 mEq/L, from 117 mEq/L to 126 mEq/L, in 24 hours.

During the process of repleting serum sodium, it is critical for providers to identify and address the underlying cause of hyponatremia. There are three categories of hyponatremia depending on the patient’s volume status: hypovolemic, hypervolemic, euvolemic [15]. In the setting of infection, euvolemic hyponatremia is most common [15]. Volume status should be assessed clinically (skin turgor, capillary refill time, edema, lung sounds, jugular venous distention [JVD]). This patient had no edema or JVD, had normal skin turgor, and had normal capillary refill time, suggestive of euvolemia. A normal volume status, as in this case, is indicative of SIADH. Further diagnostic workup includes complete blood count, arterial blood gas, serum electrolytes, serum osmolarity, urine osmolality, and urine sodium concentration, as appropriately performed here to diagnose SIADH. It is also important to review home medications, as thiazide diuretics, narcotics, antiepileptics, and antidepressants can also lead to hyponatremia [15]. None of these factors were identified in this patient. If an infectious cause is suspected, workup can include urine culture, wound culture, blood culture, and chest X-ray. All of these diagnostic steps were appropriately performed and led to treatment with empiric broad-spectrum antibiotic coverage, fluid resuscitation, and cautious sodium correction. This treatment successfully corrected the patient's hyponatremia and AKI to baseline within a few days, though prolonged treatment was needed for complete resolution of infection and respiratory symptoms.

## Conclusions

Our report highlights an unusual case of severe hyponatremia in a young, previously healthy patient presenting with severe sepsis. Despite immunocompetence and minimal risk factors, the patient presented with dangerously low sodium concentration, likely due to SIADH in the setting of sepsis secondary to skin abscess and pneumonia. As demonstrated by the patient’s serum sodium trends after presentation, sodium levels were carefully corrected from 117 mEq/L to 126 mEq/L over 24 hours and up to 137 mEq/L in 48 hours, thus adhering to safe correction thresholds. The patient's AKI also rapidly resolved due to appropriate broad-spectrum antibiotics and fluid resuscitation. While the severe sepsis and respiratory symptoms necessitated a prolonged ICU stay, appropriate diagnosis and treatment of hyponatremia in the ED prevented immediate mortality. The severity of the patient’s sodium imbalance was a key factor in recognizing the severity of his disease and the emergent nature of necessary treatment. The immediate actions to correct hyponatremia and treat SIADH helped the patient return to baseline after his infection resolved. In the ED, rapid recognition and correction of hyponatremia is critical in limiting hospital length of stay, morbidity, and mortality.
